# Effect of wearables with behavioral prompts on physical and cognitive outcomes in community-dwelling older adults: preliminary results of a pilot randomized controlled trial

**DOI:** 10.3389/fspor.2026.1768808

**Published:** 2026-08-25

**Authors:** Kar Yin Lee, Savannah Siew, Tat Lee Teo, Kuang Chua Chua, Junhong Yu, Rathi Mahendran, Iris Rawtaer

**Affiliations:** 1 Department of Psychiatry, Sengkang General Hospital, Singhealth Duke-NUS Academic Medical Centre, Singapore, Singapore; 2 Psychology, School of Social Sciences, Nanyang Technological University, Singapore, Singapore; 3 School of Engineering, Ngee Ann Polytechnic, Singapore, Singapore; 4 Yeo Boon Khim Mind Science Centre, National University of Singapore, Singapore, Singapore; 5 Mind Care Clinic, Farrer Park Medical Center, SBF Medical Suites, Singapore, Singapore

**Keywords:** behavioral intervention, cognitive decline, frailty, physical exercise, randomised controlled trial, technology

## Abstract

**Introduction:**

The global rise in life expectancy has driven research into aging-related vulnerabilities, particularly cognitive frailty. Physical activity interventions have been identified as a protective factor, and technology, such as wearable devices, may enhance engagement. This pilot RCT explores mobile phone applications and smartwatch-based interventions to promote physical activity and improve cognitive health in older adults.

**Methods:**

This single-blind pilot RCT enrolled 53 adults aged 60–85 in Singapore through convenience sampling. Participants were randomized (1:1) into an intervention group receiving daily activity prompts or a control group with no prompts. Primary outcomes like physical activity levels, neurocognitive scores, and physical frailty scores were assessed at baseline, 3 months, and 6 months. We hypothesize that the intervention group will demonstrate higher physical activity levels, leading to improved cognitive and physical outcomes at end point.

**Results:**

This study found no significant differences in physical activity levels between the intervention and control groups. However, the intervention group showed significant improvements in balance and fall risk, with a greater reduction in Timed Up and Go scores from T1 to T2 (*p* = .026). For neurocognitive outcomes, the intervention group demonstrated significant improvement in the Colour Trail Test over time, whereas the control group's performance declined from T1 to T2 (*p* = .044). At T3, the intervention group experienced a greater decline in performance in the Digit Span Backwards test from T1 to T3 compared to the control group (*p* = .014).

**Discussion:**

This study highlights the benefits of digital physical activity interventions for the physical and cognitive health of older adults. However, the findings underscore the complexities of behaviour change, suggesting that adherence significantly influences outcomes.

**Clinical Trial Registration:**

ClinicalTrials.gov, identifier NCT04692974

## Introduction

The pace of global population aging is accelerating. According to the World Health Organization, by 2030, one in six people will be aged 60 or over, increasing to 2.1 billion by 2050. This demographic shift is accompanied by a growing burden of cognitive decline, physical frailty, and loss of independence, posing a substantial challenge to health and social care systems (1, 2). Cognitive frailty is broadly understood as the co-occurrence of cognitive impairment and physical frailty in the absence of major neurocognitive disorder. It was initially defined by an international consensus group convened by the International Academy on Nutrition and Aging (I.A.N.A) and the International Association of Gerontology and Geriatrics (I.A.G.G) in 2013 (3). Although the concept has evolved to encompass a wider spectrum of cognitive vulnerability, the close relationship between cognitive decline and physical frailty remains clinically important (4). These two conditions frequently co-exist and are increasingly understood to share common underlying mechanisms, including vascular dysfunction, chronic low-grade inflammation, and metabolic dysregulation (5–7). Reduced physical activity, which is often a consequence of both cognitive impairment and frailty, may further exacerbate biological vulnerability by diminishing neuroplasticity, impairing cardiovascular and metabolic health, and accelerating functional decline (8).

Observational studies consistently demonstrate that higher levels of physical activity are associated with a reduced risk of cognitive decline and dementia (9–11). Furthermore, randomized controlled trials provide some evidence of benefit that aerobic and resistance exercise in older adults produce moderate improvement in cognitive performance compared with control interventions. Benefits have been observed across multiple domains, including executive function, global cognition, episodic memory, and attention (12). Beyond cognition, the benefits of exercise extend to physical function and frailty-related outcomes (13, 14). Resistance training has been shown to improve key components of physical frailty, including handgrip strength, lower limb strength, balance, gait speed, and stair-climbing performance (15, 16). The cognitive and physical benefits of exercise are mediated by biological mechanisms such as improved cerebral perfusion, increased neurotrophic factors, and reduced neuroinflammation (17, 18).

However, translating these benefits into sustained behaviour change among older adults remains challenging, with adherence often limited by motivational, environmental, and health-related barriers (19, 20). In addition to these behavioral challenges, the relationship between physical activity and physical and cognitive outcomes is shaped by the broader clinical context of aging. Older adults frequently live with multiple chronic conditions and are exposed to polypharmacy, both of which have been independently associated with poorer physical and cognitive function (21–23). Sensory impairments, including hearing and vision loss, are also common and have been linked to reduced physical activity levels and accelerated cognitive decline (24–26). Together, these interrelated factors may influence baseline functioning, engagement with interventions, and responsiveness to behavioral strategies. This underscores the complexity of implementing and evaluating lifestyle interventions in real-world older adult populations.

In recent years, wearable activity-tracking technologies have emerged as widely available tools that facilitate real-time self-monitoring of physical activity with minimal supervision. Consumer devices including wrist-worn smartwatches such as Apple Watch and fitness bands by Garmin and Fitbit can capture metrics such as step count, movement intensity, and heart rate with reasonable validity (27–31). Their increasing accessibility and uptake among older adults make them a promising approach to support behaviour change. Existing studies showed that approximately 80% of community-dwelling older adults in Europe and North America report access to smartphones and other forms of information and communication technology. For example, data from a German cohort indicate that 80.7% of adults aged 65 years and older use digital technology, including smartphones, computers and tablets (32). Similarly, an Australian cohort study found that digital technology use was highly prevalent among adults aged 55–74 years, with 87% reporting broad use of digital devices (33). These findings suggest that the adoption of wearable devices among older adults is both feasible and encouraging, although it is influenced by factors such as age, educational attainment, socioeconomic status, health status, and digital literacy.

Despite growing interest in wearable technology, relatively few randomized controlled trials have examined whether wearable-based interventions produce sustained improvements in physical activity compared with usual care in real-world settings. A 2020 systematic review identified 23 eligible trials and reported that interventions incorporating activity trackers were associated with a moderate improvement in physical activity (34). Similarly, in the Active for Life trial, 243 older Australians were randomized to a 12-week web-based programme incorporating computer-tailored advice and Fitbit activity trackers (35). Participants receiving the combined tailored intervention and Fitbit demonstrated a 59% greater increase in physical activity compared with controls. However, relatively few studies have explored downstream cognitive outcomes attributable to increased activity in older populations. As a result, the potential of wearable technology as a scalable preventive strategy against cognitive decline remains incompletely understood.

Against this backdrop, we conducted a pilot randomized controlled trial to investigate whether the provision of wearable activity trackers paired with behavioral prompts could effectively promote physical activity, and thereby favorably influence physical and cognitive outcomes in older populations. We hypothesized that the intervention group would achieve higher levels of physical activity, which in turn would lead to improvement in cognitive and physical outcomes compared with the control group.

## Methodology

### Study design

This study employed a single-blind randomized controlled trial (RCT) design to investigate the effectiveness of smartwatches paired with mobile phone applications in promoting physical activity among older adults. This study was approved by the National University of Singapore's Institutional Review Board on 17 August 2020 (NUS-IRB Ref. No.: H-20-038). The conduct of the study was guided by the Good Clinical Practice Principles and the Human Biomedical Research Act (2015) in Singapore. Written informed consent was obtained from participants. An information sheet was provided, and they were given ample time to consider their participation before signing the consent document. The Mini-Mental State Examination (MMSE) was administered to confirm that participants had the cognitive capacity to provide consent at the time it was obtained.

### Study procedure

Detailed information regarding the study's methodology has been previously published (36). Block randomization was carried out to ensure an equal number of participants in each treatment arm, either the intervention or the control group. Assessors were blinded to participants’ treatment assignments while administering the questionnaires. Group assignments and the subsequent installation of the mobile application and smartwatch occurred after baseline assessments were completed.

Baseline information (T1) was gathered on participants’ demographics, neurocognitive data, physical activity, and physical frailty. Follow-up assessments of cognitive and physical outcomes were conducted at 3 months (T2) and 6 months (T3). The 6-month follow-up enabled us to evaluate the sustainability of the intervention and to rule out novelty effects. The trial was conducted after the main period of nationwide COVID-19 lockdowns, but during the study, there were intermittent periods of enhanced concerns and re-implementation of restrictions when community case counts spiked, and local public health advisories recommended that older adults limit non-essential outings.

### Participants

The first participant was recruited on 15th December 2020. Participants were recruited from an existing cohort study, the Community Health and Intergenerational (CHI) study or via word-of-mouth referrals from sources such as senior activity centres or memory clinics. The CHI study is a cohort study investigating biopsychosocial factors of aging among community-dwelling older adults aged 60 years and above in Singapore (37). Eligibility criteria for this study included participants aged between 60 and 85 years who did not have any severe cognitive impairments as assessed by the Mini-Mental State Examination. Exclusion criteria were participants who were already engaging in vigorous physical exercise, did not own an Android phone that could support at least a 6.0 Operating System or had medical contraindications for physical exercise.

**Figure 1:**
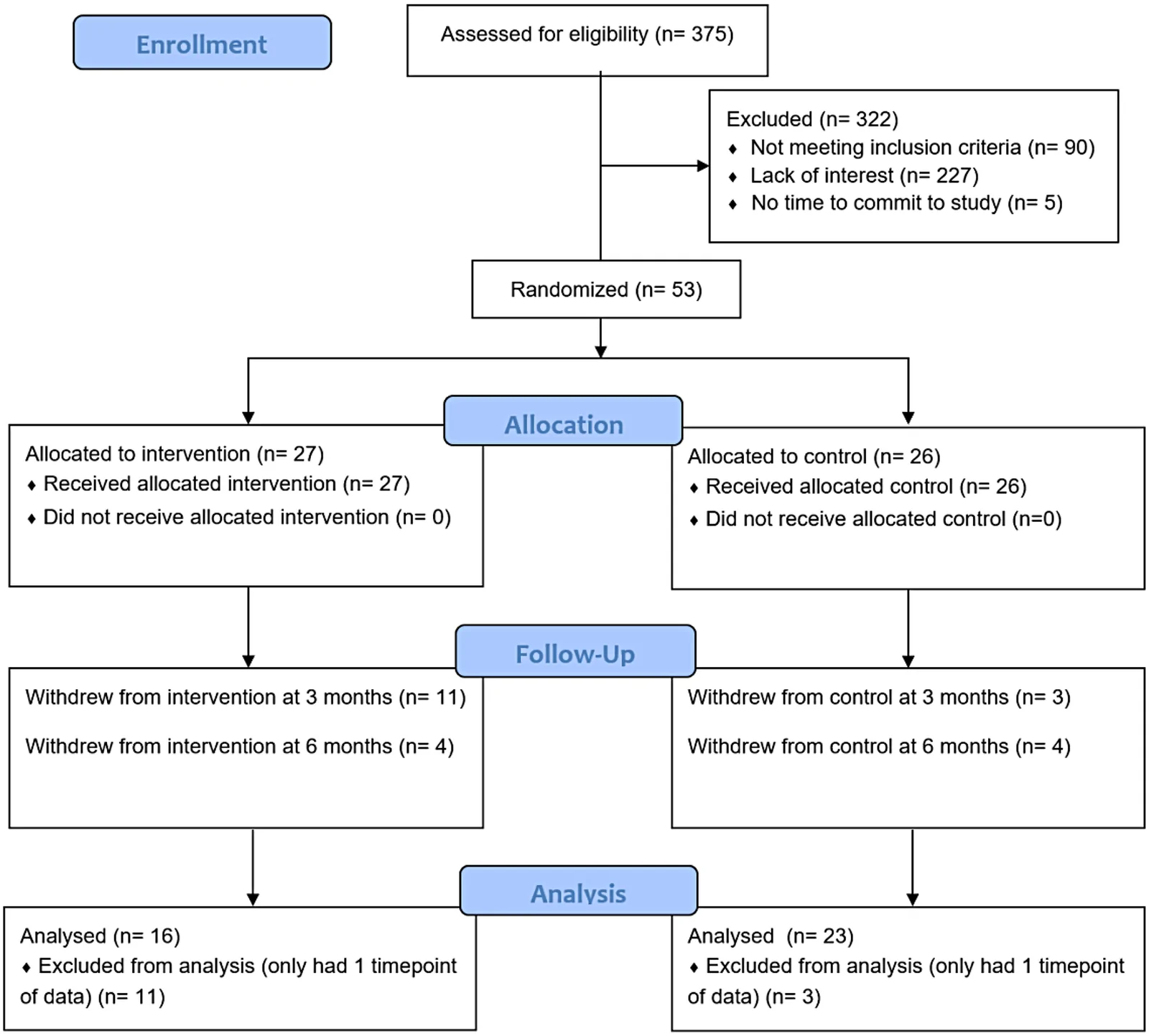
Consort flow diagram.

Given a hypothesized treatment effect size of 0.4 according to previous similar studies, it was estimated using G*Power that a total sample size of 42 was required to detect a significant within-between interaction with a power of.80 (assuming a = .05; correlation between repeated measures = .50; nonsphericity correction = 1). After considering potential dropout rates and publication value, a total sample size of 60 was required. However, due to COVID-19-related challenges outlined above, only 53 participants met eligibility criteria and agreed to be enrolled in the study. Table 1 shows the baseline sociodemographic characteristics of our sample. The intervention and control groups did not have statistically significant differences in any of the sociodemographic variables. The mean age of the population was 69.17 years, with 12.90 years of education and the majority of the participants were from the Chinese racial group. Majority were retired and resided in public housing. 14 participants withdrew at the 3-month mark while 8 participants withdrew at 6 months. After excluding those without at least two timepoints of measurements, which is necessary for the statistical analysis, the final analyzed sample had 39 participants. Figure 1 reports the CONSORT flow diagram.

**Table 1:** Sociodemographic characteristics of our sample and comparison between group assignments.

Psychosocial-demographic characteristics	Sample, (*n* = 53)	Intervention, (*n* = 27)	Control, (*n* = 26)
	Mean (SD)/%		
Age	69.17 (4.96)	69.96 (4.97)	68.35 (4.91)
Sex			
Female	73.6	66.7	80.8
Male	26.4	33.3	19.2
Race			
Chinese	96.2	96.3	96.2
Malay	0	0	0
Indian	1.9	3.7	0
Others	1.9	0	3.8
Years of education	12.90 (3.74)	12.74 (3.48)	13.06 (4.06)
Employment			
Retired	69.8	66.7	73.1
Employed	30.2	33.3	26.9
Housing type			
1–3 room HDB	15.1	18.5	11.5
4–5 room/EA/EM HDB	50.9	59.3	42.3
Private housing	34.0	22.2	46.2

HDB = housing development board, which are government-subsidized housing, EA = executive apartments, EM = executive masionette.

#### Intervention group

The intervention involved notification prompts aimed at promoting physical activity. Participants were advised to engage in 150 min of moderate intensity exercise each week and to achieve a daily step count of 7,500 steps. Participants in the intervention group were provided with smartwatches, which they used alongside a mobile application throughout the study. The smartwatch tracked physical activity by monitoring the number of steps taken and the duration of moderate physical activity, as inferred from heart rate data. Heart rate and step counts were recorded whenever participants wore the watch, which was typically during waking hours, while the watch was charged overnight. Participants were required to log their physical activity by activating the tracker on the smartwatch. If they did not achieve the recommended activity levels, they received notification prompts through both the mobile application and the smartwatch. The application also provided access to a map of nearby workout locations. If participants failed to reach their target step counts, they received two prompts—one at midday and another in the evening, stating “You have not reached the minimum steps today”. Additionally, if they did not meet their weekly goals for moderate-intensity exercise, another prompt was issued midweek, stating “You have not reached your minimum amount of moderate exercise today”. The notification prompts were designed to encourage increased physical activity and were supplemented with information about available workout spaces and facilities nearby. The mobile application also included safety reminders to help participants exercise carefully and safely. The safety reminder included the statements “Be careful not to over-exert yourself as you will be at risk of injuries”, “Please wear appropriate footwear and clothing for exercising”, and “Hydrate regularly”. Users could select between English and Mandarin language settings within the application.

#### Control group

The control group utilized the smartwatch solely as a tracking device to collect heart rate and step count data. However, they were not able to view this information, as their devices were modified to display only the time and they did not receive any notification if they did not hit the target number of steps or minutes of moderate intensity exercise.

### Measures

#### Physical activity

The primary outcome of this study was physical activity, and subjective physical activity data were gathered using the International Physical Activity Questionnaire short form (IPAQ) (38), which records the hours spent in vigorous and moderate physical activity, walking, and sitting over the past seven days. This questionnaire has previously been employed in studies assessing physical activity within our local population (39). Additionally, a translated version of the IPAQ demonstrated adequate validity and reliability among a comparable older adult population (40).

#### Neurocognitive outcomes

A comprehensive neurocognitive assessment test battery (NCA) was administered to evaluate cognitive outcomes as secondary outcomes and all tests and test scores used can be found in Table 2. The NCA included the following tests: the Rey Auditory Verbal Learning Test (41), Digit Span (Forward and Backward) subtest from the Wechsler Adult Intelligence Scale III (42), the Colour Trails Test (43), Block Design subtest from the Wechsler Adult Intelligence Scale III (42), and Semantic Verbal Fluency.

**Table 2:** List of neurocognitive assessment tests and their cognitive outcomes.

Neurocognitive assessment	Test scores	Cognitive ability
Rey Auditory Verbal Learning Test *Recall a list of words presented aloud over five learning trials followed by an interference list of words*	Total learning *Sum of scores of all five learning trials* Delayed recall *Number of words accurately recalled after 30 min* Delayed recognition *Sum of target words correctly recognized, and distractor words correctly rejected*	Verbal and learning memory Delayed verbal and learning memory Recognition memory
Digit span *Listened to a series of numbers and immediately recall them*	Forward *Recalled series of numbers in the same order* Backward *Recalled series of numbers in the backwards order*	Working memory span Manipulation of information in working memory
Colour Trails Test *Quickly connect a series of numbers printed within coloured circles in a specific order for each trial, (1) sequentially from 1 to 25, (2) sequentially from 1 to 25 but alternating between pink and yellow circles*	Interference effect *Difference in completion time between trial 1 and 2* Mean response of completion time *Average completion time of both trials 1 and 2* Total errors *Total number of errors across both trials*	Sequencing and divided attention
Wechsler's Block Design *Arrange blocks with different coloured patterns to form a designated pattern shown*	Total score *Based on both accuracy and time taken to complete each stage*	Visuospatial ability
Semantic Verbal Fluency *Say aloud the names of as many different types of animals as possible within 1 min*	Total score *Number of different animals mentioned*	Verbal fluency

#### Physical outcomes

Another secondary outcome, physical outcomes, was assessed using the FRAIL questionnaire, which addresses Fatigue, Resistance, Ambulation, Illness, and Weight Loss. Scores range from 0 to 5, with each question scored as Yes (= 1) or No (= 0). A FRAIL score of 0 indicates normal aging, scores of 1 to 2 indicate pre-frailty, and scores of 3 to 5 indicate frailty. Despite its simplicity, the FRAIL scale has been validated as a reliable screening tool for frailty in various older adult community samples (44, 45), including our local population (46).

Additionally, physical performance measures related to frailty were collected, as recommended by the Asia-Pacific Clinical Practice Guidelines (47). These measures included (1) grip strength, (2) gait speed, (3) the Timed Up and Go (TUG) test, and (4) a component of the Short Physical Performance Battery (SPPB). To avoid redundancy, only the time taken to rise from a chair and return to a seated position five times from the SPPB was measured (48).

### Statistical analysis

All analyses were performed in R 4.4.1. Bootstrapped independent t-test and Fisher's exact test were used to calculate the group differences in the sociodemographic and moderating variables between the two groups depending on whether the variables were continuous or categorical, respectively. Statistical significance was set at *p* < .05. Bootstrapping was performed with 5,000 bootstrap samples.

Effects of the digital physical activity intervention relative to the control condition on physical activity, neurocognitive and physical outcomes were estimated using models containing the main effects of group (intervention and control), time (baseline, 3 months, 6 months), and their interaction. Control variables included depression, levels of motivation, and sleep quality. These were measured by the Geriatric Depression Scale, the Barriers Self-efficacy Scale, and the Pittsburgh Sleep Quality Index, respectively. Multiple comparison correction following the Benjamini-Hochberg false discovery rate correction was applied within each outcome category. *post-hoc* sensitivity analysis estimates that a minimum effect size of 0.52 can be detected with 80% power.

## Results

**Figure 2:**
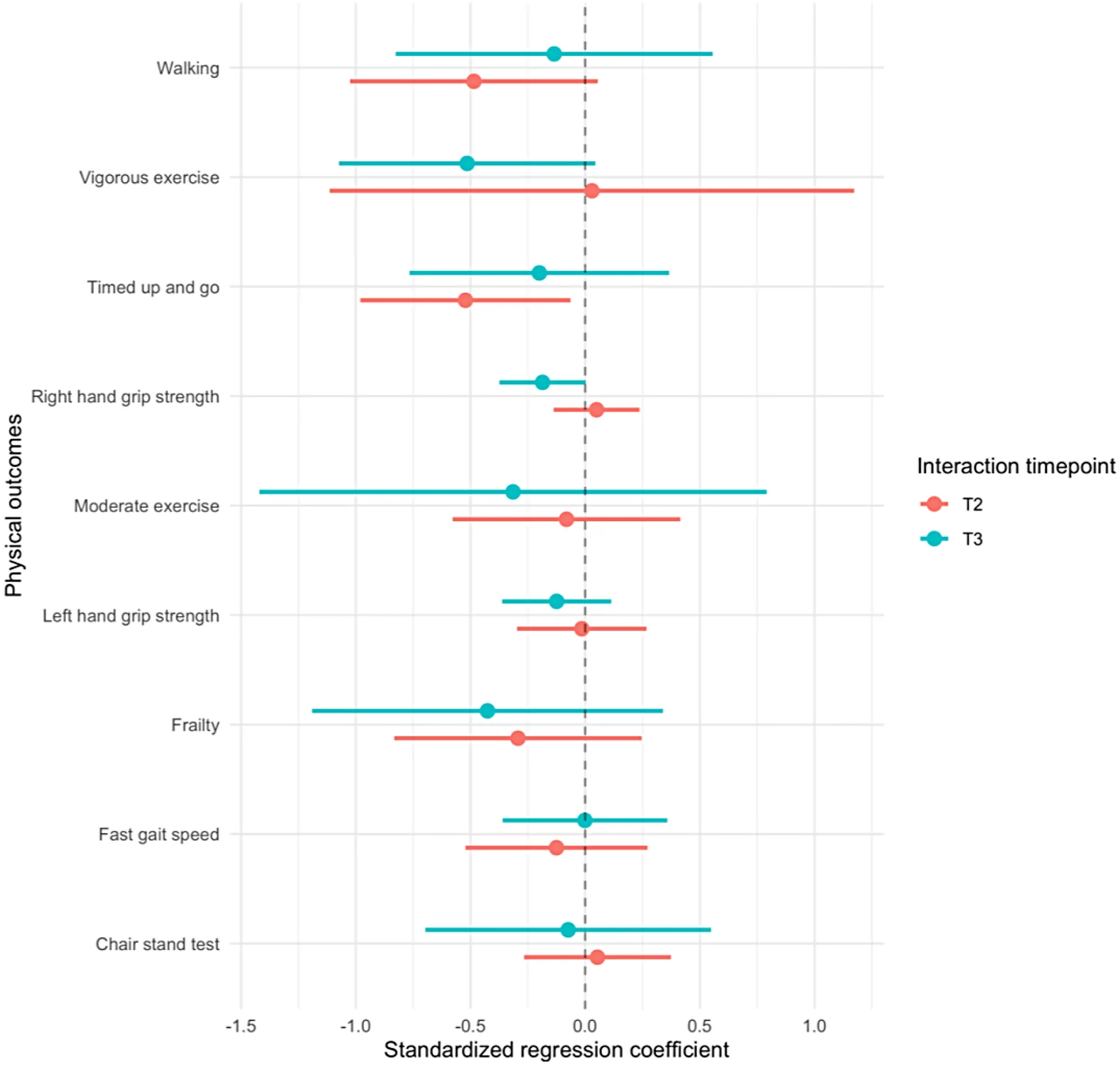
Standardized coefficient of group by time interaction for all physical outcomes. The standardized regression coefficients are represented by the dots within their respective lines that correspond to their 95% confidence intervals. The reference timepoint is the baseline measurement.

For the interaction effect between group x time, this was not significant for the amount of physical activity. In terms of physical outcomes, TUG was a significant physical outcome variable for this interaction effect (see Figure 2). The intervention group's TUG score improved more significantly than the control group from T1 to T2 but did not survive multiple comparison correction (*β* = −0.52, *p* = .026, *p_adj_ =* .153).

**Figure 3:**
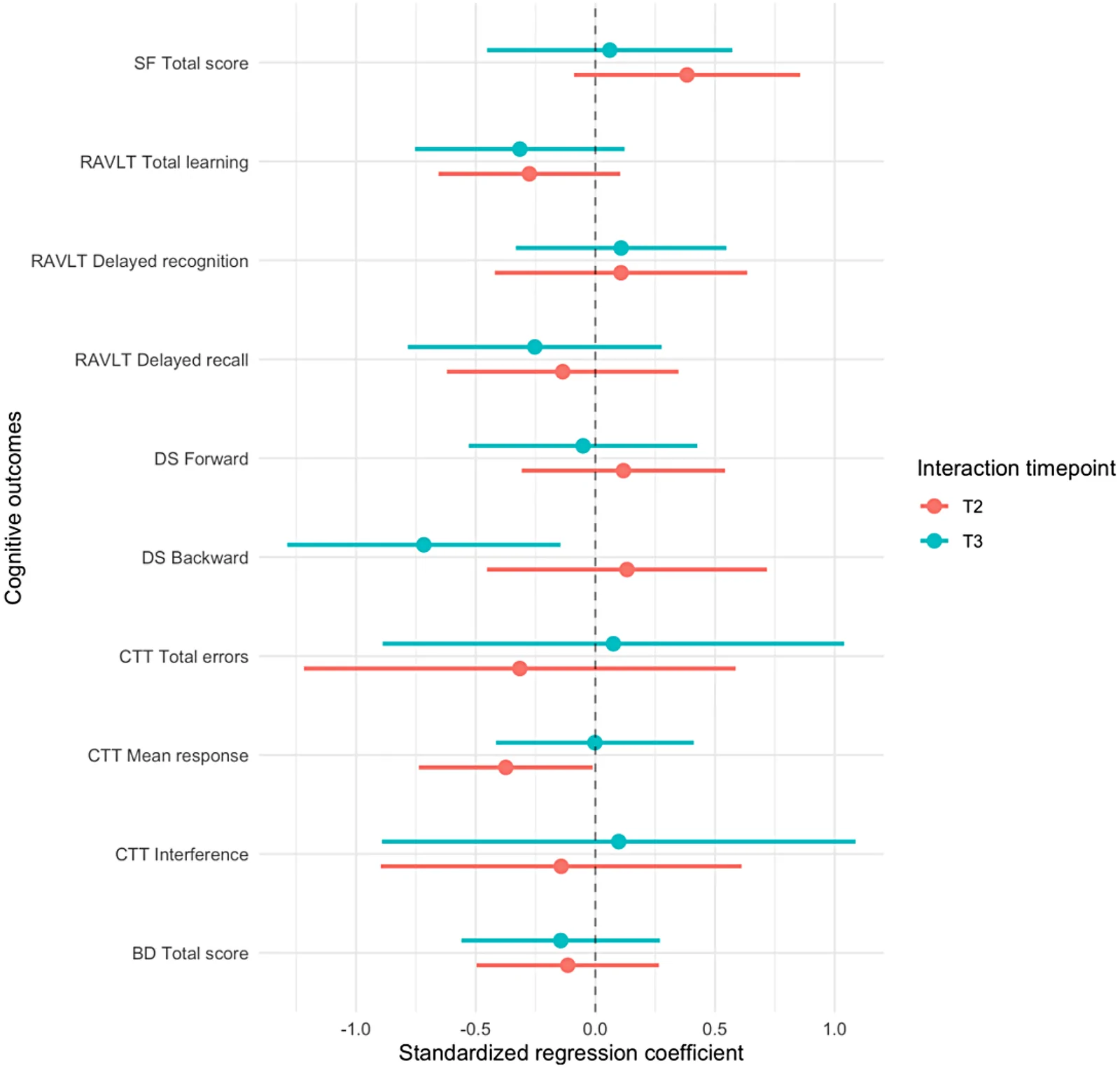
Standardized coefficient of group by time interaction for all cognitive outcomes. The standardized regression coefficients are represented by the dots within their respective lines that correspond to their 95% confidence intervals. The reference timepoint is the baseline measurement. SF, Semantic Fluency, RAVLT, Rey Auditory Verbal Learning Test, DS, digit span, CTT, Color Trails Test, BD, Block Design.

In terms of neurocognitive outcomes, the mean response of the colour trail test (CTT) and digit span backwards (DSB) was significant (see Figure 3). The intervention group's mean response on the CTT improved over time while the control group's score worsened over time from T1 to T2 (*β* = −0.37, *p* *=* .044*, p_adj_ =* .436). The intervention group also had a significantly worse drop in DSB performance from T1 to T3 than the control group (*β* = −0.72, *p* = .014*, p_adj_ =* .139), although both did not survive multiple comparison correction.

## Discussion

This pilot study aimed to investigate the effectiveness of smartwatches paired with mobile phone applications in promoting physical activity and enhancing cognitive and physical outcomes among older adults. Contrary to our initial hypothesis, the results indicated no significant differences in physical activity levels between the intervention and control groups. Despite the intervention not leading to a measurable increase in overall physical activity, there is significant improvement in the physical indicator of balance and fall risk of the intervention group at the 3-month mark. For the neurocognitive aspect, there is significant improvement in the intervention group's divided attention scores at the 3-month mark. However, the intervention group showed significantly worse performance in the working memory task at the 6-month mark. It is important to note that the results did not survive multiple comparison correction and the findings should be interpreted as preliminary rather than confirmatory. Despite the intervention group receiving prompt notifications designed to encourage activity, these prompts did not translate into measurable increases in physical activity over the 6-month follow-up period.

Several factors may contribute to these unexpected findings. Although the recruitment for this study happened after the main bulk of COVID-19-related restrictions and lockdowns, there were multiple periods of enhanced concern regarding the situation when spikes in cases occurred. Given that older adults are the most vulnerable population, these spikes in cases led to advisories for them to limit their movements and activities outside. Thus, the COVID-19 pandemic has had profound effects on lifestyle behaviours, particularly physical activity levels among older adults. A systematic review of 25 studies that examined physical activity levels in people older than 60 years found a significant reduction in physical activity during periods of restriction during the pandemic (49). Lockdowns, safety concerns, and social distancing measures likely limited outdoor activity opportunities for both the intervention and control groups. This external factor complicates the interpretation of our findings, as the environmental influences on physical activity were beyond the control of the study design.

Beyond external environmental influences, the substantial missing objective physical activity data and reliance on self-reported measures represent a critical limitation that warrants careful consideration in this pilot RCT. Step counts and activity metrics were frequently not synchronised because participants did not consistently enable Bluetooth or activate tracking functions on their devices despite multiple reminders. This led to substantial missing objective data. Due to incomplete objective data, physical activity was assessed primarily using the IPAQ. Although widely used, the IPAQ has recognized limitations, particularly self-report bias and measurement bias (50, 51). Participants may struggle to accurately recall the frequency, duration, and intensity of activities over the preceding week. This measurement limitation prevents definitive conclusions about whether the intervention truly failed to increase physical activity. It is therefore important to recognize the barriers to wearable device adoption among older adults. These challenges are often multifactorial, encompassing individual, technological and psychosocial factors (52, 53). Limited digital literacy, low confidence in using new technology and device fatigue are common difficulties in this population (54, 55). Practical technological issues like remembering to charge devices, navigating applications, enabling Bluetooth pairing and ensuring cloud synchronization could further hinder sustained use. Future studies should account for these barriers when designing larger-scale trials, with particular emphasis on simplified device interfaces, structured onboarding, and ongoing technical support to enhance adherence and long-term engagement.

Despite the lack of measurable differences in reported activity levels, the intervention group showed improvement in functional mobility as assessed by the TUG test. Several factors may account for this finding. The TUG test is a widely recognized tool for assessing functional mobility and fall risk in older adults and is sensitive to subtle changes in neuromuscular function and coordination (56, 57). Even small behavioral adjustments not captured by self-report measures may result in measurable improvements in TUG performance (58, 59). In this pilot study, although overall activity did not differ significantly based on IPAQ data, wearable prompts may have influenced movement patterns rather than total duration or intensity of activity. Participants may have interrupted prolonged sitting more frequently, engaged in short bouts of light activity, or walked with greater awareness and confidence. These brief movements of standing, short walks and transitional movements may improve lower limb activation, balance, and gait speed, thereby contributing to improved functional mobility (60–62).

CTT is an assessment tool designed to evaluate sustained and divided attention. In particular, the mean response time taken measures participants’ ability to set-switch while completing the task. Improvement in divided attention on the CTT in this pilot study might reflect the cognitive demands associated with using wearable technology. Self-monitoring activity, responding to prompts, and adjusting behaviour require planning, attentional control, and executive regulation. Walking while monitoring feedback may also introduce a mild dual-task component, indirectly training attentional flexibility. Even small increases in structured, goal-directed behaviour can enhance frontal network engagement (63, 64). While evidence in older adults remains limited, studies in younger populations have demonstrated associations between higher aerobic fitness and better sustained attention, as well as between physical activity and metacognitive performance (65, 66). The potential relationship between physical activity and sustained attention may be explained by the cardiovascular fitness hypothesis, which suggests that improved cardiovascular fitness mediates cognitive benefits (67). Additionally, physical activity stimulates the release of brain-derived neurotrophic factor (BDNF), a protein that supports neuronal growth and survival, further enhancing cognitive performance (68). The combined effects of increased arousal, elevated BDNF levels, and enhanced cerebral blood flow serve as key mechanisms underlying the positive impact of exercise on sustained attention.

Working memory manipulation was assessed by the DS backwards task. Interestingly, we saw a drop in working memory performance in the intervention group at the 6-month mark. One possible explanation relates to cognitive load. The use of wearable devices requires ongoing monitoring, responding to prompts, and behavioral self-regulation. While such engagement may enhance divided attention and executive flexibility, it may also increase mental demands. Working memory is especially sensitive to interference and mental fatigue (69, 70). Sustained self-monitoring could therefore contribute to reductions in working memory efficiency (71). Additionally, individual variability may also have influenced the results. Working memory performance is sensitive to sleep quality, stress, physical fatigue and motivation (72). If follow-up assessments coincided with periods of tiredness or reduced motivation, performance could decline independent of true cognitive change. Furthermore, improvements in working memory are more consistently associated with gains in aerobic fitness and prefrontal efficiency than with light-intensity activity. Evidence from systematic reviews suggests that moderate-intensity, multi-component, or mind–body exercises performed regularly over several months are most likely to enhance working memory in older adults (73). If the intervention did not substantially increase moderate-to-vigorous physical activity, it may not have produced the level of cardiovascular adaptation that supports improvement in this domain. Behavioral prompts that altered movement patterns without meaningfully enhancing cardiorespiratory fitness may not have been sufficient to produce working memory gains.

## Strengths and limitations

This pilot RCT has several strengths. Its randomized controlled design enhances internal validity and reduces selection bias. The study examined both physical and cognitive outcomes, allowing a more comprehensive evaluation of the intervention's potential multidimensional effects. The use of a wearable device with behavioral prompts reflects a pragmatic, scalable approach that is highly relevant to community-dwelling older adults. In addition, the inclusion of objective functional measures alongside standardized neuropsychological assessments strengthens the clinical relevance of the findings.

There are some limitations inherent in our study design and lessons from this pilot study that must be acknowledged and improved on in future studies. Objective measurements of physical activity are one such limitation, as our study relied on the IPAQ. The physical activity questionnaire, which relies on a 7-day recall period, may not accurately capture habitual physical activity levels. The short recall period may not reflect typical activity patterns, especially in populations with variable routines. As a self-reported measure it is also susceptible to recall bias, potentially contributing to the lack of observed differences in our study. Our study initially tried to include objective measures of physical activity using a wearable device such as steps taken and minutes of moderate exercise. However, technological and user-related factors led to several issues that impeded consistent data acquisition. For example, the wearable devices required a stable Bluetooth connection to sync collected data with participants’ smartphones for these to be stored. Many participants were not aware of frequent disconnections and were not able to troubleshoot issues at home after set-up was completed at the research site, leading to incomplete data synchronization and, thus, data collection. Participants also reported being less familiar with Bluetooth connections and usage in comparison with internet connection tools like Wi-Fi. The key takeaway is to use wearable sensors and systems that are either cloud-connected or capable of storing data offline until retrieval.

Another limitation of this study is the differential dropout rate between groups. By the 3-month follow-up, 41% of participants in the intervention group had withdrawn compared to only 12% in the control group. While this level of attrition is concerning, *post-hoc* analysis (see Supplementary Material) showed that within the intervention group, those who dropped out did not differ from those who completed on any baseline characteristic, suggesting that dropout was not systematically related to who was doing better or worse at the start. Notably, the three participants who withdrew from the control group had significantly higher TUG scores at baseline, meaning they were more physically impaired than those who stayed. The final analyzed subgroups at both 3 and 6 months remained balanced on age, sex, and education, which supports the validity of the between-group comparisons. Nonetheless, we cannot rule out that unmeasured factors influenced who dropped out, particularly in the intervention group, where the demands of using the wearable device and mobile application may have discouraged continued participation among less technology-comfortable individuals. This should be considered when interpreting the findings, and future studies should incorporate more structured onboarding and technical support to reduce intervention-related dropout.

Additionally, the study faced challenges in recruiting participants during the COVID-19 pandemic, and fewer than the *a priori* required sample size of 60 participants were recruited. This may have led to an underpowered study that failed to detect meaningful differences between the groups. Furthermore, the dropout rate, particularly amid the COVID-19 pandemic, may have skewed our results. The pandemic posed significant challenges to recruitment and participant retention, as many older adults faced increased health concerns and social isolation, which could have influenced their willingness or ability to engage consistently with the intervention. Given that we have found some significant cognitive and physical benefits among the intervention group in this pilot study, future research with a larger sample size could better support the use of such interventions. Continued investigation into the optimal design and delivery of such interventions is essential to fully realize their potential benefits. Exploring the long-term effects of such interventions, beyond the initial novelty phase, could provide further insights into their potential for promoting sustained physical activity in older adults.

## Conclusion and future directions

This pilot study demonstrates both the promise and challenges of wearable device interventions for older adults. While technical barriers prevented adequate measurement of physical activity changes, the observed improvements in physical function suggest that behavioral prompts may beneficially influence how older adults move, even if total activity level remains unchanged. The mixed cognitive findings highlight the domain-specific nature of cognitive responses to physical activity and the need for carefully designed interventions targeting specific cognitive outcomes.

Future research should prioritize: (1) developing and validating wearable devices specifically designed for older adults with enhanced usability and accuracy; (2) implementing comprehensive technical support systems to maximize device adherence and data completeness; (3) incorporating both objective and subjective activity measures alongside qualitative assessments of how participants modify their daily activities; (4) examining mechanisms linking behavioral prompts to functional improvements, including movement quality and functional exercise integration; (5) conducting adequately powered trials with longer intervention durations to assess sustained effects on multiple cognitive domains; and (6) identifying subgroups of older adults most likely to benefit from wearable device interventions based on baseline characteristics, technology literacy, and support systems.

Despite the measurement challenges encountered, this pilot study provides valuable insights into the complex relationships between wearable device interventions, physical activity, and functional outcomes in older adults, informing the design of future definitive trials in this important area.

## Data Availability

The raw data supporting the conclusions of this article will be made available by the authors, without undue reservation.
