# Supplementary material for: Effect of wearables with behavioral prompts on physical and cognitive outcomes in community-dwelling older adults: preliminary results of a pilot randomized controlled trial
Source: Front Sports Act Living. 2026 Aug 25;8:1768808. doi: 10.3389/fspor.2026.1768808 (PMC13552123; doi:10.3389/fspor.2026.1768808)
Supplement: Supplementary file 1 [file Table1.docx]

Supplementary Material

**Table A**

*Baseline Characteristics of Completers and Dropouts by Group*

|  | **Intervention** | | | **Control** | | |
| --- | --- | --- | --- | --- | --- | --- |
|  | Completers  (*n* = 16) | Dropouts  (*n* = 11) | *p* | Completers  (*n* = 23) | Dropouts  (*n* = 3) | *p* |
| **Demographics** | | | | | | |
| Age, *M* (*SD*) | 70.25 (5.01) | 69.55 (4.66) | .709 | 68.26 (4.79) | 69.00 (4.90) | .832 |
| Years of education, *M* (*SD*) | 13.19 (2.30) | 12.09 (4.50) | .407 | 13.28 (4.17) | 11.33 (0.94) | .449 |
| Female, % | 56.2 | 81.8 | .231 | 78.3 | 100.0 | 1.000 |
| **Clinical and Physical Outcomes** | | | | | | |
| FRAIL score, *M* (*SD*) | 0.25 (0.75) | 0.36 (0.64) | .685 | 0.22 (0.41) | 0.33 (0.47) | .791 |
| TUG (seconds), *M* (*SD*) | 9.35 (2.18) | 10.02 (2.75) | .475 | 8.26 (1.78) | 11.25 (0.96) | .018* |
| **Neurocognitive Outcomes** | | | | | | |
| Digit Span Forward, *M* (*SD*) | 10.19 (2.79) | 10.55 (2.54) | .736 | 10.14 (2.58) | 11.67 (1.89) | .325 |
| Digit Span Backward, *M* (*SD*) | 7.38 (2.15) | 7.64 (1.23) | .715 | 7.00 (1.95) | 8.00 (2.16) | .435 |
| CTT Trial 1 (seconds), *M* (*SD*) | 48.06 (19.11) | 51.82 (17.56) | .598 | 47.09 (17.27) | 36.67 (4.78) | .317 |
| CTT Trial 2 (seconds), *M* (*SD*) | 95.94 (21.65) | 96.55 (19.57) | .944 | 94.91 (23.40) | 78.33 (13.60) | .240 |
| Block Design, *M* (*SD*) | 31.31 (9.99) | 31.45 (9.95) | .966 | 31.55 (11.26) | 40.67 (11.09) | .214 |
| Semantic Fluency, *M* (*SD*) | 16.00 (3.04) | 18.36 (3.39) | .075 | 18.71 (6.68) | 18.33 (2.36) | .935 |
| RAVLT Total Learning, *M* (*SD*) | 50.06 (7.73) | 50.27 (9.55) | .952 | 49.05 (11.52) | 52.00 (3.56) | .667 |
| RAVLT Delayed Recall, *M* (*SD*) | 11.00 (1.94) | 10.64 (3.75) | .738 | 9.77 (2.75) | 12.67 (0.47) | .095 |
| RAVLT Recognition, *M* (*SD*) | 14.19 (0.95) | 14.18 (1.19) | .993 | 14.64 (0.71) | 14.00 (0.00) | .128 |

*Note.* Continuous variables compared using bootstrapped independent *t*-tests (5,000 samples); categorical variables compared using Fisher's exact test. TUG = Timed Up and Go; CTT = Colour Trails Test; RAVLT = Rey Auditory Verbal Learning Test. * *p* < .05.

**Table B**

*Baseline Comparability of Analysed Subgroups at T2 and T3*

|  | **T2** | | | **T3** | | |
| --- | --- | --- | --- | --- | --- | --- |
|  | Intervention  (*n* = 16) | Control  (*n* = 23) | *p* | Intervention  (*n* = 12) | Control  (*n* = 19) | *p* |
| Age, *M* (*SD*) | 70.25 (5.01) | 68.26 (4.79) | .224 | 69.92 (5.68) | 67.84 (4.33) | .259 |
| Years of education, *M* (*SD*) | 13.19 (2.30) | 13.28 (4.17) | .936 | 13.25 (2.13) | 12.68 (4.04) | .672 |
| Female, % | 56.2 | 78.3 | .174 | 66.7 | 84.2 | .384 |

*Note.* Continuous variables compared using bootstrapped independent *t*-tests (5,000 samples); categorical variables compared using Fisher's exact test. No statistically significant between-group differences were observed at either timepoint (all *p* > .05).
